# Functional expression of chloride channels and their roles in the cell cycle and cell proliferation in highly differentiated nasopharyngeal carcinoma cells

**DOI:** 10.14814/phy2.12137

**Published:** 2014-09-11

**Authors:** Weiyuan Huang, Mei Liu, Linyan Zhu, Shanwen Liu, Hai Luo, Lianshun Ma, Haibo Wang, Ruiling Lu, Xiaoxue Sun, Lixin Chen, Liwei Wang

**Affiliations:** 1 Department of Physiology, Medical College, Jinan University, Guangzhou, China; 2 Department of Pharmacology, Medical College, Jinan University, Guangzhou, China

**Keywords:** Cell cycle, cell proliferation, chloride channels, nasopharyngeal carcinoma, regulatory volume decrease

## Abstract

We previously demonstrated that the growth of the poorly differentiated nasopharyngeal carcinoma cells (CNE‐2Z) was more dependent on the activities of volume‐activated chloride channels than that of the normal nasopharyngeal epithelial cells (NP69‐SV40T). However, the activities and roles of such volume‐activated chloride channels in highly differentiated nasopharyngeal carcinoma cells (CNE‐1) are not clarified. In this study, it was found that a volume‐activated chloride current and a regulatory volume decrease (RVD) were induced by 47% hypotonic challenges. The current density and the capacity of RVD in the highly differentiated CNE‐1 cells were lower than those in the poorly differentiated CNE‐2Z cells, and higher than those in the normal cells (NP69‐SV40T). The chloride channel blockers, 5‐nitro‐2‐(3‐phenylpropylamino) benzoic acid (NPPB) and tamoxifen inhibited the current and RVD. Depletion of intracellular Cl^−^ abolished the RVD. The chloride channel blockers reversibly inhibited cell proliferation in a concentration‐ and time‐dependent manner, and arrested cells at the G0/G1 phases, but did not change cell viability. The sensitivity of the three cell lines to the chloride channel blockers was different, with the highest in poorly differentiated cells (CNE‐2Z) and the lowest in the normal cells (NP69‐SV40T). ClC‐3 proteins were expressed in the three cells and distributed inside the cells as well as on the cell membrane. In conclusion, the highly differentiated nasopharyngeal carcinoma CNE‐1 cells functionally expressed the volume‐activated chloride channels, which may play important roles in controlling cell proliferation through modulating the cell cycle, and may be associated with cell differentiation. Chloride channels may be a potential target of anticancer therapy.

## Abstract (precis)

In this paper, we demonstrated that the volume‐activated chloride channels were involved in regulating CNE‐1 cells proliferation and cell cycle progress. Thus, volume‐activated chloride channels may be a potential target of anticancer therapy.

## Introduction

Nasopharyngeal carcinoma affects predominantly young population in Southeast Asia (Hong et al. 2013). The mechanism of nasopharyngeal carcinoma genesis is complex, involving aberration of a large variety of pathways (Chou et al. 2008). Although great progress has been made, the exact mechanism underlying the development of nasopharyngeal carcinoma remains unknown.

Recently, ion channels have been suggested to be associated with cell proliferation and/or cancer development (Ghiani et al. 1999; Okada et al. 2009; Xu et al. 2010; Ding et al. 2012; Zhu et al. 2012). The expression and activity of chloride channels have been demonstrated to be important in keeping some characteristics of cells, such as anchorage‐dependent growth (Voets et al. 1995; Al‐Nakkash et al. 2004; Sun et al. 2012). Our previous study suggests that the activities and expression of chloride channels is upregulated in the poorly differentiated nasopharyngeal carcinoma cell (CNE‐2Z) and plays more important roles in regulation of cell growth in the cancerous cell than in its counterpart, the immortalized normal nasopharyngeal epithelial cell (NP69‐SV40T, derived from the normal nasopharyngeal epithelium) (Zhu et al. 2012).

Nasopharyngeal carcinoma is a nonlymphomatous and squamous cell neoplasm that occurs in the epithelial lining of the nasopharynx and exhibits various degrees of differentiation (Wei and Sham 2005). Different from the CNE‐2Z cell line, which was derived from a patient with poorly differentiated squamous nasopharyngeal carcinoma (Gu et al. 1983), the CNE‐1 cell line was derived from a patient suffering from highly differentiated squamous nasopharyngeal carcinoma (Chinese Academy of Medical Sciences, Zhongshan Medical College 1978). The biological features are different between the two cell lines. CNE‐1 cells are spindle or irregular and Epstein–Barr virus negative (Masmoudi et al. 2007; Hippocrate et al. 2011), whereas CNE‐2Z cells are polygon and Epstein–Barr virus positive (Vasef et al. 1997). Compared with CNE‐1 cells, CNE‐2Z cells display a far more irregular and confused intermediate filament organization structure, with lower content of keratin and more variety of keratin subtypes (Ma 1993), and are more invasive (Kong 1992). The doubling time of cell growth in CNE‐1 cells is around 20–24 h, while that in CNE‐2Z cells is about 16–18 h (Wu et al. 2006).

As mentioned above, we have demonstrated previously that chloride channels play more important roles in controlling cell proliferation in poorly differentiated nasopharyngeal carcinoma CNE‐2Z cells than in normal cells. In this study, we used the whole‐cell patch‐clamp technique, cell image analysis methods, MTT assay, and flow cytometry to investigate the functional activities and roles of volume‐activated chloride channels in the highly differentiated nasopharyngeal carcinoma CNE‐1 cells.

## Materials and Methods

### Cell culture

The highly differentiated (CNE‐1) and poorly differentiated (CNE‐2Z) human nasopharyngeal carcinoma cells and the immortalized normal human nasopharyngeal epithelial cells (NP69‐SV40T) were cultured with the RPMI 1640 medium containing 10% new‐born calf serum (Gibco, Grand Island, NY), 100 unit/mL penicillin G, and 100 *μ*g/mL streptomycin at 37°C in a humidified atmosphere of 5% CO_2_. The CNE‐2Z cell line was kindly provided by Professor Weiping Tang (Department of Pathology, Guangdong Medical College, China). The CNE‐1 and NP69‐SV40T cell lines were obtained from Hunan Xiangya Type Culture Collection (Hunan, China).

### Electrophysiology

Whole‐cell chloride currents were recorded using the patch‐clamp technique as described by us previously Chen et al. (2002), Bai et al. (2010) with an EPC‐7 amplifier (HEKA, Darmstadt, Germany). The patch‐clamp pipettes showed a 4–5 MΩ resistance when filled with the pipette solution. The liquid junction potential was corrected when the pipette entered the bath and the access resistance was compensated. The whole‐cell capacitance was determined by adjusting and minimizing the capability transients in response to a 20 mV voltage step. Cells were held at the Cl^−^ equilibrium potential (0 mV) and then stepped in sequence to ±80, ±40, 0 mV repeatedly, with a 200 ms duration for each step and 4 s intervals between steps. Data were sampled at 3 kHz and collected through a laboratory interface (CED 1401, Cambridge, UK). The current density was determined by dividing the whole‐cell current with the membrane capacitance in each individual cell.

### Anion substitution experiments

When the hypotonicity‐induced current reached the peak, NaCl (70 mmol/L) in the 47% hypotonic solution was replaced by equimolar NaI, NaBr, or sodium gluconate. The anion permeability ratios (*P*_X_/*P*_Cl_) relative to that of Cl^−^ were calculated using the modified Goldman‐Hodgkin‐Katz equation, *P*_X_/*P*_Cl_ = {[Cl^−^]_n_exp(−Δ*V*_rev_*F*/*RT*) − [Cl^−^]_s_} / [X^−^]_s_, where [Cl^−^]_n_ and [Cl^−^]_s_ stand for the concentration of Cl^−^ in the normal and substituted bath solutions respectively, [X^−^]_s_ is the concentration of the substituted anion, Δ*V*_rev_ is the difference of the reversal potentials for Cl and X, and *F*,* R,* and *T* are the Faraday constant, gas constant, and absolute temperature respectively (Chen et al. 2002).

### Measurements of cell volume

Cell images were captured at 30 sec intervals by a CCD digital camera (Mono CCD625, Leica, Wetzlar, Germany). The equation *V* = (4/3) × *π *× (*d*/2)^3^ was used to calculate the cell volume, where *d* is the cell diameter. The regulatory volume decrease (RVD) was calculated as follows: RVD (%) = (*V*_max_ − *V*_min_) / (*V*_max_ − V_0_) × 100%, where *V*_0_, *V*_max_, *V*_min_ represents the cell volume in the isotonic solution, the peak volume in hypotonic solutions and the volume before returning to the isotonic solution, respectively. Experiments were performed at 20–24°C.

### Cell proliferation assay (MTT assay)

Cells were cultured in 96‐well culture plates for 14 h and treated with the media containing different reagents for 24–72 h. 10 *μ*L stock solution of 3‐(4, 5‐dimethyl‐thiazol‐2‐yl)‐2, 5‐diphenyl tetrazolium bromide (MTT, 5 mg/mL) was added to each well, and the cells were incubated at 37°C for 4 h. The medium was removed, and 100 *μ*L DMSO was added into each well to dissolve the purple formazan crystals. The absorbance (expressed as optical density, OD) was recorded at 570 nm by an automated plate reader (Model 680; BIO‐RAD, Berkeley, CA).

### Cell cycle analysis

Cell cycle distribution was determined using a flow cytometer (EPICS XL; Coulter Co., Hialeah, FL) as previously described Wang et al. (2002), Chen et al. (2007). Cells were collected, fixed with pre‐cold (at −20°C) 70% ethanol, washed twice with PBS, incubated in a staining buffer (50 *μ*g/mL propidium iodide, 100 *μ*g/mL RNase and 0.1% Triton X‐100 in PBS) at 37°C for 30 min, and analyzed with the flow cytometer.

### Immunofluorescence

For immunofluorescence, cells were plated on 6‐mm round glass coverslips and cultured in 24‐well plates for 24 h before fixation with paraformaldehyde (4%) and sucrose (0.12 mol/L) in PBS. Cells were permeabilized with Triton X‐100 (0.3% in PBS), blocked with 10% normal sheep serum (Sigma‐Aldrich, St. Louis, MO) in PBS for 45 min, treated with the rabbit anti‐ClC‐3 primary antibody (1:50; Alomone Labs, Jerusalem, Israel) overnight at 4°C and incubated in FITC conjugated goat anti‐rabbit secondary antibody (1:50; Proteintech Group, Inc., Chicago, IL) and DAPI (5 *μ*g/mL; Beyotime Institute of Biotechnology, Haimen, China) at room temperature for 30 min. Finally, the coverslips with cells were inverted onto glass slides, sealed with nail varnish and examined by a confocal microscope (C1 Si; Nikon, Tokyo, Japan).

### Solutions and chemicals

The patch‐clamp pipette solution contained (in mmol/L): 70 N‐methyl‐D‐glucamine‐chloride, 1.2 MgCl_2_, 10 HEPES, 1 EGTA, 140 D‐mannitol, and 2 ATP. The isotonic bath solution contained (mmol/L): 70 NaCl, 0.5 MgCl_2_, 2 CaCl_2_, 10 HEPES and 140 D‐mannitol. The osmolarity of pipette and isotonic bath solutions was adjusted to 300 mOsmol/L with D‐mannitol. Forty‐seven percent hypotonic solution (160 mOsmol/L) was obtained by omitting 140 D‐mannitol from the isotonic bath solution. The pH of the pipette and bath solutions was adjusted to 7.25 and 7.4, respectively. The chloride channel blockers, 5‐nitro‐2‐(3‐phenylpropylamino) benzoic acid (NPPB) and tamoxifen, were dissolved in dimethyl sulfoxide and methanol at the concentration of 100 and 50 mmol/L, respectively, and diluted to final concentration with bath solutions. All chemicals were purchased from Sigma (Sigma‐Aldrich).

### Statistics

Data were expressed as mean ± standard error (number of observations) and were analyzed using the Student's *t* test and ANOVA. Statistical significance was defined as *P *<**0.05. All experiments were repeated at least three times.

## Results

### Functional expression of volume‐activated chloride channels in CNE‐1 cells

**Figure 1.**
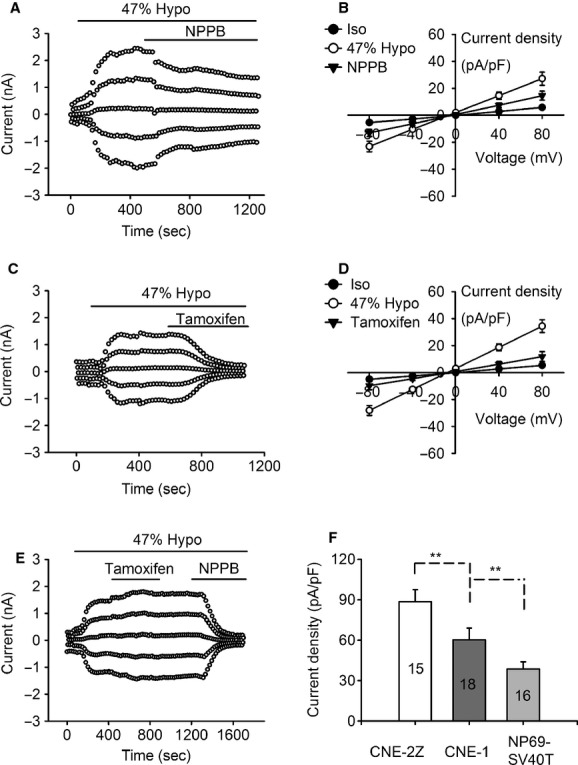
Activation of hypotonicity‐induced chloride currents and inhibition of the currents by the chloride channel blockers NPPB and tamoxifen in CNE‐1 cells. Cells were held at 0 mV and then stepped in sequence to ±80, ±40 and 0 mV repeatedly. Forty‐seven percent hypotonic challenges (Hypo) activated a chloride current which was inhibited by 100 *μ*mol/L NPPB (A & B) and 20 *μ*mol/L tamoxifen (C & D). (E) shows the inhibitory effect of NPPB (100 *μ*M) on the tamoxifen (20 *μ*mol/L)‐insensitive current. (F) presents the comparison of the hypotonicity‐activated currents between CNE‐2Z, CNE‐1 and NP‐69‐SV40T cells (*n* = 15, 18,16 respectively). Data in B, D and F are mean ± SE of 8–18 cells. *P <**0.05, **P <**0.01.

As shown in Fig. 1, the basal current recorded in the isotonic solution was small with a mean value of 12.3 ± 1.5 pA/pF at +80 mV (*n *=**18) in CNE‐1 cells. When exposed to 47% hypotonic solution, a large current was activated. Similar to that recorded in CNE‐2Z cells and NP69‐SV40T cells (Zhu et al. 2012), the hypotonicity‐activated current did not exhibit obvious outward rectification, with the reversal potential (−1.6 ± 0.2 mV, *n *=**18) close to the calculated equilibrium potential for Cl^−^ (Fig. 1A–E). However, the current density at +80 mV (60.3 ± 8.6 pA/pF, *n *=**18) was smaller than that in the poorly differentiated CNE‐2Z cells (88.5 ± 8.9 pA/pF, *n *=**15, *P *<**0.01) and larger than that in the normal NP69‐SV40T cells (38.5 ± 5.5 pA/pF, *n *=**16, *P *<**0.01) (Fig. 1F).

The chloride channel blocker NPPB (100 *μ*mol/L) inhibited the hypotonicity‐activated current in CNE‐1 cells (Fig. 1A and B). The current was decreased by 59.5 ± 12.2% at +80 mV and 57.5 ± 13.5% at −80 mV (*n *=**8; *P *<**0.05, vs. control).

The chloride channel blocker tamoxifen (20 *μ*mol/L) could also inhibit the hypotonicity‐activated current, but the inhibitory efficiency varied among the cells (Fig. 1C–E). Some (five out of eight cells) were sensitive to tamoxifen, with the inhibition of 70.5 ± 20.0% at +80 mV and 72.9 ± 19.7% at −80 mV (*P *<**0.01, vs. control), but the others were not sensitive to tamoxifen. Further study indicated that the tamoxifen‐insensitive current could be inhibited by 100 *μ*mol/L NPPB (Fig. 1E). Similar to that in CNE‐1 cells, the heterogeneity in the response to tamoxifen was also observed in CNE‐2 cells and NP69‐SV40T cells.

In the anion permeability experiments, 70 mM NaCl in the 47% hypotonic solution was replaced by equimolar NaI, NaBr, or sodium gluconate. Analysis of the data indicated that the anion permeability of the chloride channels in CNE‐1 cells was I^−^ > Br^−^ > Cl^−^ > gluconate, with the permeability ratios (*P*_X_/*P*_Cl_) of 1.12 ± 0.02 for I^−^ (*n *=**6), 1.10 ± 0.02 for Br^‐^ (*n *=**6), and 0.53 ± 0.01 for gluconate (*n *=**6).

### Regulatory volume decrease (RVD) in CNE‐1 cells and the involvement of the chloride channels in RVD

**Figure 2.**
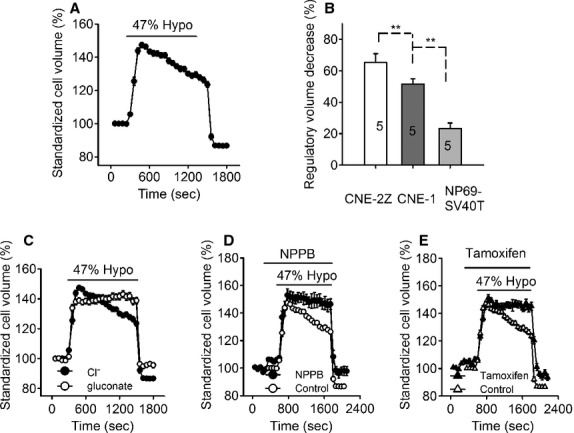
Hypotonicity‐induced RVD and the effects of depletion of intracellular Cl^−^ and extracellular application of the chloride channel blockers NPPB and tamoxifen on RVD in CNE‐1 cells. Exposure to a 47% hypotonic solution swelled CNE‐1 cells and induced a regulatory volume decrease (RVD) (A). RVD in CNE‐2Z cells was the largest and that in NP‐69‐SV40T cells was the smallest with that in CNE‐1 in the middle (B, five experiments). Depletion of intracellular Cl^−^ by incubation the cells in the Cl^−^‐free solution (substitution of NaCl with equimolar sodium gluconate) for 2 h (C), or extracellular application of 100 *μ*mol/L NPPB (D) or 20 *μ*mol/L tamoxifen (E) abolished the hypotonicity‐induced RVD. Data in the figures are mean ± SE of 16–39 cells in 3–5 experiments. *P <**0.05, **P <**0.01.

As shown in Fig. 2A, exposure to 47% hypotonic bath solution swelled the cells and induced a regulatory volume decrease. The cell swelling appeared in about 1 min and reached a peak in 2–5 min, with an increase of 46.7 ± 8.8% in cell volume (39 cells in five experiments, *P *<**0.01). The cell volume was then decreased gradually toward the control level although the cells were still bathed in the hypotonic solution. The cells were recovered by 51.6 ± 3.3% in volume 20 min after application of hypotonic challenges. Further analysis indicated that the RVD process varied among the cells. The RVD in highly differentiated CNE‐1 cells (51.6 ± 3.3%) was smaller than that of poorly differentiated CNE‐2Z cells (65.3 ± 5.6%, 38 cells in five experiments, *P *<**0.01), but higher than that in the normal nasopharyngeal epithelial NP69‐SV40T cells (23.2 ± 3.6%, 49 cells in five experiments, *P *<**0.01) (Fig. 2B).

Further experiments indicate that the outflow of chloride is an important RVD mechanism in CNE‐1 cells. The cells were bathed in the Cl^−^‐free isotonic solution for 2 h, in which the sodium chloride was replaced by equimolar sodium gluconate. This treatment could deplete both the extracellular and intracellular chloride. The results showed that, after 2 h incubation in the Cl^‐^‐free isotonic bath solution, the exposure of cells to the Cl^−^‐free hypotonic bath solution could still swell the cells, but could hardly induce a regulatory volume decrease (Fig. 2C).

The hypotonicity‐induced RVD could be inhibited by the chloride channel blockers, NPPB and tamoxifen in CNE‐1 cells. As shown in Fig. 2D, addition of NPPB (100 *μ*mol/L) in the isotonic bath solution increased slightly the cell volume. Application of 47% hypotonic solution containing 100 *μ*M NPPB could still swell the cells, but the RVD was inhibited by 60.3 ± 3.0% (16 cells in three experiments; *P *<**0.01, vs. control). Similar to the effects of NPPB, tamoxifen (20 *μ*mol/L) swelled the cells in the isotonic condition and inhibited the hypotonicity‐induced RVD by 80.2 ± 2.6% (29 cells in 4 experiments, *P *<**0.01, Fig. 2E).

### Association of volume‐activated chloride channels with cell proliferation

**Figure 3.**
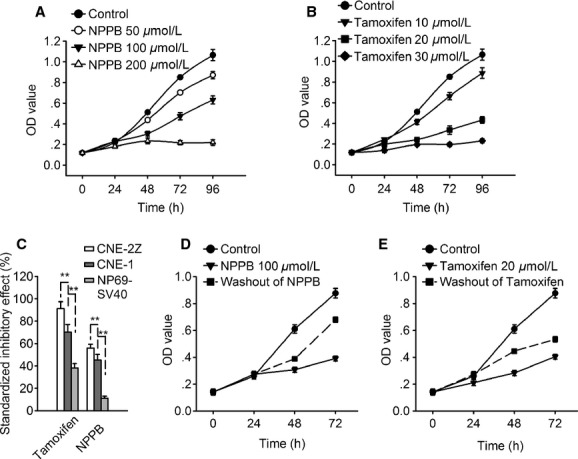
Inhibitory effects of the chloride channel blockers NPPB and tamoxifen on CNE‐1 cell proliferation. Relative cell number was detected by the MTT assay and expressed as the optical density (OD value). (A) and (B) present the inhibitory effects of 100 *μ*mol/L NPPB and 20 *μ*mol/L tamoxifen on cell proliferation. (C) presents the comparison of the inhibitory effects of NPPB and tamoxifen between CNE‐2Z, CNE‐1, and NP‐69‐SV40T cells (D) and (E) shows the release of cells from the inhibitory effects of NPPB and tamoxifen by the washout of the inhibitors. Data in the figures are mean ± SE of four experiments.

Addition of the chloride channel blocker NPPB or tamoxifen in the medium inhibited CNE‐1 cell growth in a concentration and time‐dependent manner (Fig. 3A and B). The treatment of CNE‐1 cells with 100 *μ*mol/L NPPB or 20 *μ*mol/L tamoxifen for 72 h inhibited cell proliferation by 45.3 ± 5.1% (*n *=**4, *P *<**0.01) or 70.3 ± 6.6% (*n *=**4, *P *<**0.01), respectively. Compared with the inhibitory effects of NPPB and tamoxifen at 72 h on the poorly differentiated CNE‐2Z cells (55.9 ± 3.5% and 91.2 ± 6.2%, *n *=**4, *P *<**0.01) and the normal NP69‐SV40T cells (11.2 ± 1.8% and 38.3 ± 3.8%, *n *=**4, *P *<**0.01), the sensitivity of highly differentiated CNE‐1 cells to NPPB and tamoxifen was in the middle among the three cells (Fig. 3C).

Further experiments showed that NPPB and tamoxifen inhibited cell growth, but did not change cell viability in the tested concentration. Cell viability was detected by the trypan blue assay, in which the dead cells would be stained. The cells were sampled and stained with the trypan blue 24–96 h after treatments. In the tested concentration (as shown above), the ratios of dead cells in NPPB and tamoxifen‐treated groups were not significantly different from those in the control group.

The inhibitory effects of NPPB and tmoxifen on cell growth were reversible. After treated with NPPB or tamoxifen for 24 h, the cells were washed and then incubated in the control medium for 48 h. The results showed that the cell growth was significantly inhibited when NPPB or tamoxifen was present in the medium, but could be recovered once NPPB and tamoxifen were washed off (Fig. 3D and E).

### Involvement of volume‐activated chloride channels in regulation of the cell cycle

The above results showed that blockage of chloride channels suppressed cell growth, but did not significantly induce cell death, suggesting that the blockers may suppress cell proliferation by a cell death‐independent mechanism. To study further, the effects of the chloride channel blockers, NPPB, and tamoxifen, on the cell cycle were tested by the flow cytometry.

**Figure 4.**
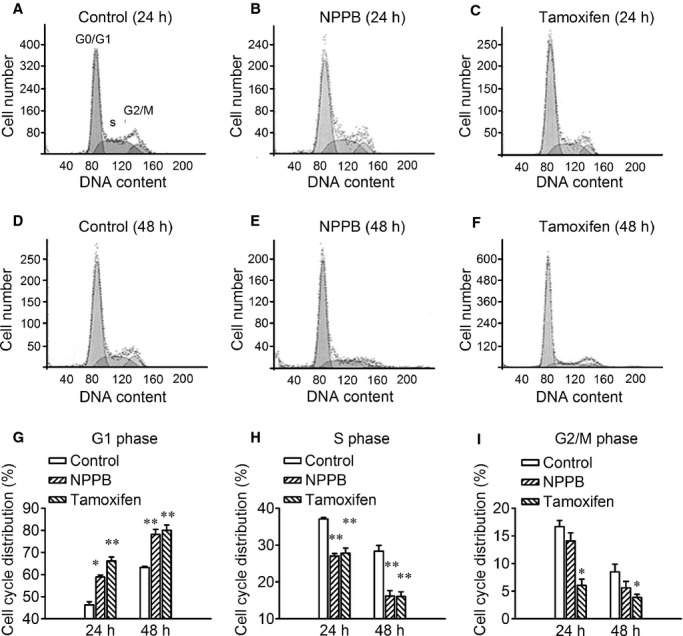
Effects of the chloride channel blockers, NPPB, and tamoxifen, on CNE‐1 cell cycle detected by the flow cytometry. (A–F) The typical cell cycle distribution of cells incubated in the medium with or without chloride channel blockers (100 *μ*mol/L NPPB or 20 *μ*mol/L tamoxifen) for 24 and 48 h. (G–I) The quantitative distribution of cells in different phases received different treatments (mean ± SE of four experiments). *P <**0.05, **P <**0.01 (vs. Control).

The results indicated that the blockers inhibited cell cycle progress by arresting the cells in the G0/G1 phases (Fig. 4). In the control cells, the population in G0/G1, S and G2/M phases was 46.3 ± 1.4, 37.1 ± 0.3 and 16.7 ± 1.1 at 24 h, and 63.2 ± 0.4, 28.4 ± 1.6 and 8.5 ± 1.4 at 48 h, respectively. Incubation of the cells in the medium containing NPPB (100 *μ*mol/L) increased the cell population in G1/G0 phases from 46.3 ± 1.4% to 58.9 ± 0.9% at 24 h (*n *=**4, *P *<**0.01), and 63.2 ± 0.4% to 78.2 ± 2.2% at 48 h (*n *=**4, *P *<**0.01). Similar to the effects of NPPB, tamoxifen (20 *μ*mol/L) increased the G1/G0 population from 46.3 ± 1.4% to 66.2 ± 1.8% at 24 h (*n *=**4, *P *<**0.01), and 63.2 ± 0.4% to 80.0 ± 2.3% at 48 h (*n *=**4, *P *<**0.01).

### Expression of ClC‐3 chloride channel proteins

**Figure 5.**
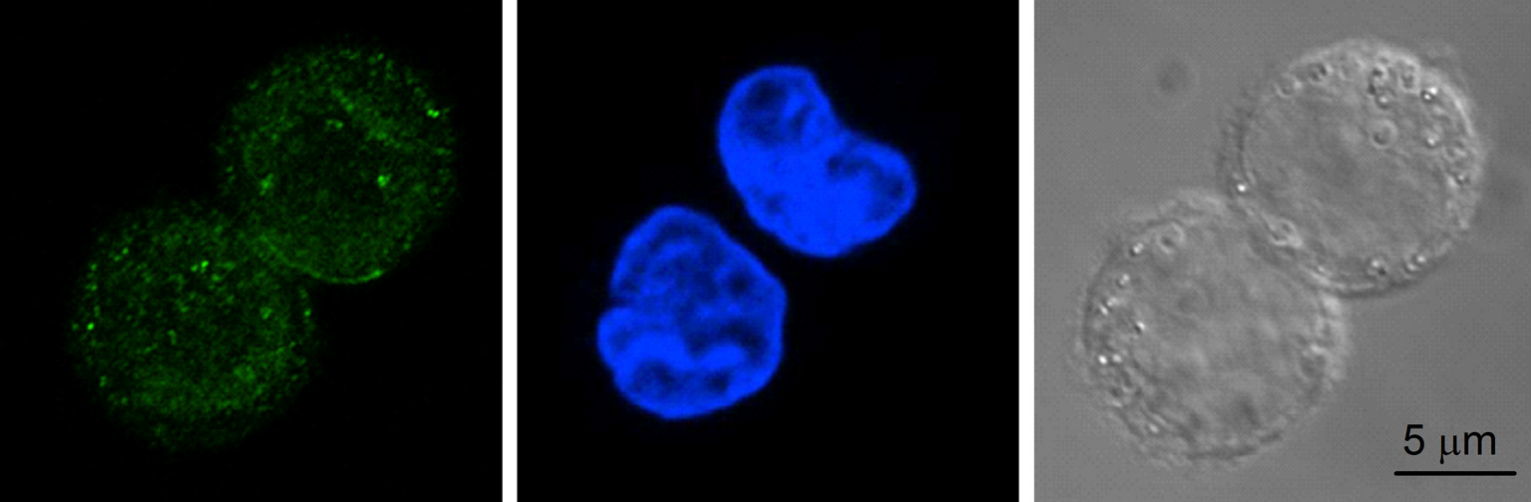
Expression of ClC‐3 chloride channel proteins detected by immunofluorescence in CNE‐1 cells. (A) ClC‐3 immunofluorescence (green). (B) The nuclei labeled by DAPI staining (blue). (C) Presents the transmitted light images of the cells.

The expression of ClC‐3 chloride channel proteins in the CNE‐1 cells was detected by the immunofluorescence. The results showed that CNE‐1 cells expressed ClC‐3 chloride channels. ClC‐3 proteins were distributed inside the cells as well as on the cell membrane, but less were located in the nucleus (Fig. 5).

## Discussion

Previously, we have study the volume‐activated chloride channels in poorly differentiated nasopharyngeal carcinoma cells (CNE‐2Z), and normal human nasopharyngeal epithelial cells (NP‐69‐SV40T) (Zhu et al. 2012). We found that chloride channel activities and the capacity of regulatory volume decrease (RVD) were upregulated in CNE‐2Z cells, compared with those in NP‐69‐SV40T cells. Other experiments including our own have shown that volume‐activated chloride channels play important roles in carcinoma cell proliferation and cell cycle progress (Chen et al. 2002; Mao et al. 2009, 2012). Different from CNE‐2Z cells, CNE‐1 cells were derived from highly differentiated nasopharyngeal carcinoma, and possessed different morphological and biological features. However, the functional activities and roles of volume‐activated chloride channels in CNE‐1 cells have not been defined yet.

In this study, we found that a current could be activated by hypotonic challenges in CNE‐1 cells. The current was reversed at a potential close to the equilibrium potential of Cl^−^, with a sequence of anion permeability of I^−^ > Br^−^ > Cl^−^ > gluconate, which was similar to that of the volume‐activated chloride current reported in other cells (von Weikersthal et al. 1999; Chung and Kim 2002; Sun et al. 2012). All these data suggest that the recorded current in CNE‐1 cells is the volume‐activated chloride current. It was further demonstrated that the current was inhibited by the chloride channel blockers NPPB and tamoxifen. NPPB inhibited both the inward and outward currents. Tamoxifen presented a thoroughly inhibitory effect in some cells, but could hardly work in some other cells. This result implies that there may be more than one subtype of volume‐activated chloride channels existing in the highly differentiated nasopharyngeal carcinoma cells (CNE‐1). The heterogeneity in the response to tamoxifen was also observed in the poorly differentiated CNE‐2Z cells and the normal NP‐69‐SV40T cells. These data suggest that the expressed subtypes of volume‐activated chloride channels are similar in the three cell lines. It was proved by us previously that ClC‐3 chloride channels were expressed in CNE‐2Z and NP‐69‐SV40T cells, and might be an important component and/or regulator of volume‐activated chloride channels (Zhu et al. 2012). In this study, it was shown that ClC‐3 chloride channels were also expressed in CNE‐1 cells, and were distributed over the cells. This distribution is different from that in CNE‐2Z cells, in which ClC‐3 channel proteins are predominantly located inside the cells (Zhu et al. 2012). The significance and the involvement of ClC‐3 chloride channels in activation of the volume‐regulated chloride current in CNE‐1 cells remain to be further studied.

As discussed above, the properties of the volume‐activated chloride current in the highly differentiated CNE‐1 cells are similar to those recorded in the poorly differentiated CNE‐2Z cells and in the normal NP‐69‐SV40T cells. However, further analysis indicates that the density of the current in CNE‐1 cells is lower than that in CNE‐2Z cells, and higher than that in NP69‐SV40T cells. This interesting finding suggests that the functional activities of volume‐activated chloride channels may be associated with cell differentiation and proliferation. It was found in our previous study that the volume‐activated chloride current was increased in the migrated CNE‐2Z cells, and the characteristics were not significantly different from those in the nonmigrated cells. In this study, we found that the volume‐activated chloride current in the poorly differentiated CNE‐2Z cells was larger than that in the highly differentiated CNE‐1 cells, but there was no significant different in the properties of the current between the two cell lines. These data imply that the expression of volume‐activated chloride channel subtypes is not significantly changed during migration and differentiation.

The involvement of chloride channels in regulation of cell proliferation in CNE‐1 cells was confirmed by our next experiments. We found that CNE‐1 cell proliferation was inhibited by the chloride channel blockers NPPB and tamoxifen. The blockers may attenuate the growth of cells in number by inducing cell death. However, our results indicated that cell viability was not significantly altered by the blockers, and the inhibitory effects of the blockers were reversible. The results suggest that the blockers may inhibit cell proliferation by suppressing cell cycle progress. This postulation is supported by our next experiment. We found that the cell population in G0/G1 phases was increased significantly by the blockage of chloride channels with NPPB or tamoxifen, indicating that the progress of the cell cycle is suppressed. The results suggest that the volume‐activated chloride channels play important roles in regulation of the cell cycle, especially in controlling cells to pass through the G1 checkpoint. Our experiments in this study also showed that the inhibitory effect of the chloride channel blockers on CNE‐1 cell proliferation was weaker than that on CNE‐2Z cells, and stronger than that on NP69‐SV40T cells. Accumulating evidence suggests that volume‐activated chloride channels may play an important role in the development of cancer (Ransom et al. 2001; Yin et al. 2007; Mao et al. 2009; Zhu et al. 2012); Chloride channels may be a potential target of anticancer therapy.

Consistent with the finding in chloride channel activities, the capacity of regulatory volume decrease (RVD) was different among the three cell lines. The hypotonicity‐induced RVD in CNE‐1 cells was weaker than that in CNE‐2Z cells, but stronger than that in NP69‐SV40T cells. Considering the difference in differentiation levels of the three cell lines, it is speculated that RVD may play important roles in cell differentiation. It was reported by us previously that the capability of RVD was cell cycle‐dependent and was involved in cell proliferation in CNE‐2Z cells (Wang et al. 2002; Chen et al. 2007). In this study, it is also proved that the activation of volume‐activated chloride channels is a key mechanism of RVD, and the outflow of chloride through volume‐activated chloride channels is required for RVD. We found that the chloride channel blockers NPPB and tamoxifen inhibited the hypotonicity‐induced RVD in CNE‐1 cells, and depletion of intracellular chloride abolished the RVD. Furthermore, we found that the volume of CNE‐1 cells was increased slightly under the isotonic condition when NPPB or tamoxifen was added to the bath. This result suggests that some of volume‐activated chloride channels are opened under isotonic conditions, and the channels play an important role in regulation of the basal cell size.

In this study, it was found that the functional activities of chloride channels and RVD capacity were upregulated in the cancerous cells especially in the poorly differentiated cancer cells. The two carcinoma cell lines were obtained from two different patients. The CNE‐2Z cell line was derived from poorly differentiated squamous nasopharyngeal carcinoma, and the CNE‐1 cell line was from highly differentiated squamous nasopharyngeal carcinoma. Normally, poorly differentiated carcinoma is more malignant and develops faster than highly differentiated carcinoma. Together with the increased activities of the volume‐activated chloride current and RVD capacity in the poorly differentiated cells, these data suggest that the change in RVD and involvement of chloride channels are more significant in poorly differentiated cancer; chloride channels play important roles in the development of cancer. It has been reported that chloride channels play important roles in regulation of chondrocyte proliferation and differentiation (Tian et al. 2010) and is required for fibroblast‐to‐myofibroblast differentiation (Yin et al. 2008). Chloride channels promote osteodifferentiation through the runt‐related transcription factor 2 (Runx2) pathway (Wang et al. 2010). Based on these data, the cells with higher activities of chloride channels should be in a higher stage of differentiation or be much easily differentiated. However, it is not the case in the CNE‐2Z cells, which have increased chloride channel activities, but are less differentiated. It seems that chloride channels lose their function to promote differentiation, but rather keep the function for enhancing proliferation and cell cycle progress in cancer cells with undefined mechanisms. Further investigation on the mechanisms may result in the development of a method that redirects cancer cells to differentiate toward normal phenotype.

In conclusion, the highly differentiated nasopharyngeal carcinoma CNE‐1 cells posses basic activities of the volume‐activated chloride channels, which can be further activated by hypotonic challenges and play important roles in controlling cell proliferation through modulating the cell cycle, and may be associated with cell differentiation. Chloride channels may be a potential target of anticancer therapy.

## Conflict of Interest

None declared.
